# Unwanted loneliness among older adults in rural areas: associated factors and guidelines for community intervention

**DOI:** 10.3389/fragi.2026.1681481

**Published:** 2026-02-05

**Authors:** A. Yurrebaso, E. Picado-Valverde, E. García-Valverde, R. Guzmán-Ordaz

**Affiliations:** Francisco de Vitoria Chair of Human Rights, Research Center on Human Rights and Public Policy (CIDH–Diversitas), University of Salamanca, Salamanca, Spain

**Keywords:** unwanted loneliness, rural aging, older adults, emotional well-being, community activities

## Abstract

**Background:**

Unwanted loneliness among older adults has become a growing public health concern, particularly in rural contexts characterized by population aging, depopulation, and limited access to services. While many interventions focus on functional and social dimensions, the subjective and emotional roots of loneliness remain insufficiently explored. This study aims to identify profiles at greater risk of loneliness and to analyze personal and community activities that may help mitigate it in highly depopulated rural municipalities.

**Methods:**

A cross-sectional quantitative study was conducted in five rural areas of western Salamanca, in municipalities with fewer than 500 inhabitants. A total of 153 individuals aged 60 and over participated. Structured interviews were carried out, including a sociodemographic questionnaire, variables related to available services, personal and community activities, and the UCLA Loneliness Scale Version 3. Data was analyzed using descriptive statistics and chi-square tests.

**Results:**

41.2% of participants reported moderate or severe levels of loneliness. Living alone, having a low educational level, and not engaging in social or leisure activities were associated with higher levels of loneliness. Significant associations were found between loneliness and variables such as living alone (p = 0.012), not talking on the phone (p = 0.024), not reading (p = 0.010), and not engaging in community activities like going out, spending time with family, or exercising (p < 0.01). The availability of services in the municipality showed no statistically significant relationship.

**Conclusion:**

In rural contexts, loneliness among older adults appears to be more strongly influenced by the quality and frequency of social relationships than by the mere availability of services. Interventions should address not only structural or functional needs but also the emotional and relational dimensions, through personalized and community-based strategies that promote overall wellbeing. It is crucial to distinguish between social isolation and subjective loneliness, and to guide policies toward the strengthening of human connection.

## 1 Introduction

Unwanted loneliness is a subjective experience that has gained increasing relevance over recent decades as one of the most pressing public health challenges, particularly among older adults. Unlike social isolation, an objectively measurable condition characterized by a lack or absence of social interactions, loneliness refers to the emotional experience of disconnection resulting from a perceived gap between the social relationships a person desires and those they actually have. This conceptualization is solidly grounded in the Cognitive Discrepancy Model proposed by Peplau and Perlman (1979), which emphasizes the role of attributional processes, social comparison, and perceived control in the emergence and persistence of loneliness.

This phenomenon becomes particularly relevant in the context of population aging. The World Health Organization (Organización Mundial de la Salud, 2021), in its Decade of Healthy Ageing 2021–2030, has emphasized the urgent need to address loneliness and social isolation as key social determinants that negatively impact the health, functionality, and quality of life of older adults. In Spain, recent data from the National Institute of Statistics (INE, 2024) show a growing trend of older people living alone, a significant risk factor for social isolation, especially in rural municipalities with fewer than 500 inhabitants, where factors such as depopulation, geographic dispersion, and limited access to services exacerbate the situation.

The scientific literature has firmly established the association between loneliness and various adverse outcomes in physical, cognitive, and emotional health. Holt-Lunstad et al. (2015) report that loneliness and social isolation may increase the risk of premature mortality by 26%, an effect comparable to classic risk factors like obesity or smoking. Other meta-analyses, such as Valtorta et al. (2016), confirm its link with cardiovascular diseases and cognitive decline. In the psychobiological field, Cacioppo and Patrick (2008) have documented how loneliness disrupts immune function, increases physiological stress, and impairs sleep quality. Moreover, Courtin and Knapp (2017) have shown that lonely older adults make greater use of health services and show lower treatment adherence. In the Spanish context, studies by Fernández-Mayoralas et al. (2019) and Rodríguez-Blázquez et al. (2022) highlight that loneliness is a key predictor of psychological distress, even more so than physical or functional health variables.

Despite the growing visibility of the issue, major challenges remain in addressing it effectively. Institutional strategies tend to focus on structural or technological solutions, such as improving access to services or promoting active aging, while giving less attention to the subjective factors underlying the experience of loneliness. Campaigns such as *Loneliness in Older People*, promoted by AGE Platform Europe (2022), call for a more holistic approach that also considers the symbolic, emotional, and cultural dimensions of social connection, especially in rural settings where community ties have weakened.

Within this framework, the present study aims to analyze the sociodemographic, personal, and contextual factors associated with subjective feelings of loneliness in older adults living in rural municipalities of Salamanca (Castile and León, Spain). A perspective grounded in the Cognitive Discrepancy Model is adopted, examining both structural variables (household size, availability of services, degree of dependency) and psychological and behavioral ones (satisfaction with relationships, frequency of social participation, and daily activities). Through a quantitative approach and the use of the UCLA Loneliness Scale Version 3, this study seeks to provide empirical evidence useful for the design of personalized interventions that are sensitive to rural contexts, promoting a more dignified, healthy, and emotionally connected aging process.

### 1.1 Loneliness through the cognitive discrepancy model

The Cognitive Discrepancy Model proposed by Peplau and Perlman (1979), Peplau and Perlman (1981) serves as the central theoretical framework for this study. This socio-cognitive perspective understands loneliness as the gap between the interpersonal relationships a person perceives they have and those they would like to have. This view emphasizes not the quantity of social contacts, but the perceived quality of those relationships and the individual’s expectations regarding them. According to this model, loneliness is a subjective, universal, and emotionally painful experience that can occur even in the presence of objectively extensive social networks.

In Spanish literature, a traditional distinction has been made between “subjective loneliness,” the emotional experience of feeling alone, and structural situations such as a lack or scarcity of social contact, which some authors have referred to as “objective loneliness.” However, in line with international terminological standards and to promote clearer understanding among non-Spanish-speaking audiences, this manuscript uses the term social isolation to refer to such structural conditions, while reserving loneliness for the subjective experience that constitutes the central focus of this study.

**FIGURE 1:**
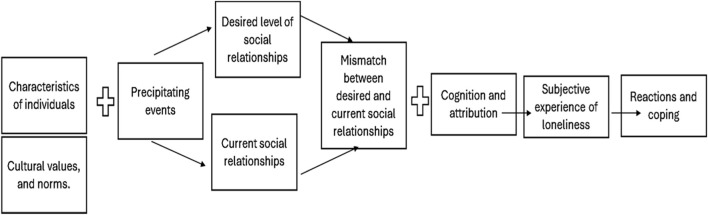
Cognitive Discrepancy Model of Loneliness (Peplau and Perlman, 1979). Source: Peplau and Perlman, p. 107.

The model, as shown in Figure 1, is structured around antecedent factors and modulating cognitive processes. Among the antecedents are predisposing and precipitating variables. Predisposing factors include personal characteristics that increase vulnerability to loneliness, such as advanced age, female gender (often associated with widowhood and caregiving roles), low educational and economic levels, minority status, and personality traits such as shyness or low self-esteem (Peplau and Perlman, 1979; Dahlberg et al., 2016; Pinquart and Sörensen, 2001). Precipitating factors, on the other hand, refer to life events that significantly alter the individual’s relational context, such as the loss of a loved one, institutionalization, retirement, or isolation caused by functional disability.

These are accompanied by three key psychological processes that influence the intensity of the loneliness experience: causal attribution, social comparison, and perceived control. Causal attribution refers to how people explain the reasons for their loneliness. When attributed to internal, unstable, and controllable factors (such as lack of initiative), individuals tend to engage in active strategies to reduce it. However, if loneliness is attributed to stable, external, or uncontrollable causes (e.g., abandonment by others), passive or resigned responses tend to emerge (Weiner, 1985). Social comparison involves evaluating one’s relational situation in comparison with that of perceived peers. Such comparisons may intensify the perception of social deficit if they are unfavorable, thus amplifying the feeling of loneliness. Lastly, perceived control refers to the extent to which an individual believes they have the capacity to influence their social situation. A high sense of control is associated with better emotional adjustment and a lower negative impact of loneliness (Cacioppo and Hawkley, 2009).

These processes explain why individuals with similar objective conditions may experience loneliness very differently, and they provide a useful framework for designing interventions. According to this model, the most effective strategies should focus on: 1) adjusting the individual’s expectations regarding the quantity and quality of social contact; 2) increasing real social contact or symbolic alternatives such as pets or technology; and 3) cognitively reframing the perceived discrepancy between desired and actual social experiences, for example, through psychological interventions.

From this perspective (Figure 1), the present study aims to empirically explore how these factors and processes manifest in a particularly vulnerable population: older adults living in rural municipalities with fewer than 500 inhabitants.

### 1.2 Sociodemographic variables and their influence on loneliness

Research on loneliness has shown that certain sociodemographic characteristics act as predisposing factors, conditioning both its onset and intensity. Age, gender, marital status, educational and economic level, and family structure have consistently been identified as variables that modulate the experience of loneliness, especially among older adults (Pinquart and Sörensen, 2001; Abellán et al., 2017; Cardona et al., 2013).

Advanced age is associated with a higher likelihood of experiencing loneliness, not only due to functional or sensory decline but also because of the progressive reduction of social networks caused by the death of close ones, retirement, or the so-called “empty nest” (Lang et al., 1998; del Barrio, 2010). Wagner et al. (1996) found that in very old age, emotional support and the number of meaningful relationships decrease significantly, heightening feelings of disconnection. Furthermore, longitudinal studies reveal a “U-shaped” curve in the prevalence of loneliness, with peaks during youth and old age (Pinquart and Sörensen, 2001), reinforcing the notion that age is a critical variable in understanding loneliness.

Gender has also been widely debated. While some studies find that women report higher levels of loneliness (Ausín et al., 2017; Aartsen and Jylhä, 2011), others conclude that men show higher scores when standardized instruments like the UCLA scale are used (Pinquart and Sörensen, 2001). This apparent contradiction may be explained by women’s greater willingness to acknowledge and verbalize feelings of vulnerability, as well as their tendency to maintain broader, though emotionally more demanding, social networks. In addition, women’s longer life expectancy means they are more often exposed to widowhood and must rely on formal or informal support networks, which are often fragile in rural areas (Borys and Perlman, 1985).

Regarding educational and economic status, multiple studies agree that higher socioeconomic levels are associated with lower levels of loneliness, as they facilitate access to resources, social activities, broader support networks, and a stronger sense of self-efficacy (Cardona et al., 2013; Rubenstein and Shaver, 1982). Older adults with low incomes not only have fewer opportunities for social interaction but may also experience impacts on self-esteem and perceived control, which in turn intensify feelings of isolation (Pinquart and Sörensen, 2001).

Marital status is another relevant variable: widowed, separated, or single individuals present a higher prevalence of loneliness compared to those who live with a partner. The loss of a significant relationship, particularly in advanced age, is not always compensated by other social networks, especially in low-density areas such as rural communities (Aartsen and Jylhä, 2011; Dahlberg et al., 2018).

In sum, sociodemographic factors act as structural determinants which, in interaction with cognitive processes and environmental conditions, shape the experience of loneliness. Therefore, their analysis is essential for identifying vulnerability profiles and designing interventions tailored to the life trajectories and individual resources of older adults.

### 1.3 Environmental factors: environment and participation

From the ecological perspective proposed by Bronfenbrenner (1979) and later models such as those of Law (1996) and Moos (1980), the physical and social environment is considered to shape participation opportunities and, therefore, directly influences the experience of loneliness. In rural settings, the lack of public transportation, scarcity of basic services, architectural barriers, and geographic isolation act as obstacles to social contact. The International Classification of Functioning, Disability and Health (ICF, World Health Organization, 2002) identifies these environmental factors as elements that can serve either as facilitators or barriers to social participation.

Participation in community activities not only reinforces the sense of belonging but also reduces isolation, improves mood, and promotes resilience. Niu et al. (2017) and Rantakokko et al. (2013) emphasize that engaging in out-of-home activities is positively correlated with subjective wellbeing and lower perceptions of loneliness. Similarly, perceived neighborhood safety, urban accessibility, and the presence of social gathering spaces are key determinants (Del Barrio et al., 2010; García and Herrero, 2006).

In the present study, participation in community social activities is analyzed as an essential dimension of the rural environment. Considering these environmental variables enables a better understanding of how loneliness manifests in contexts of high depopulation and aging and helps in designing more tailored responses to the actual needs of this population.

This theoretical framework provides a comprehensive and structured basis for analyzing loneliness through a lens that integrates structural, psychological, and contextual factors, offering a holistic approach that informs both research and future interventions.

## 2 Objectives and hypotheses

This study is grounded in the institutional recognition of loneliness as a growing problem among older adults, particularly in rural contexts marked by demographic aging, geographic dispersion, and low population density. An increase in subjective loneliness is anticipated, exacerbated by structural factors such as living in single-person or couple households, functional dependency, and difficulties accessing municipal services and community social networks.

Based on this framework, the following specific objectives are proposed.

1. To identify the sociodemographic profiles most at risk of experiencing subjective loneliness in rural settings.
2. To analyze the relationship between levels of loneliness and participation in personal (domestic, recreational) and community activities, in order to determine which activities help mitigate feelings of isolation.
3. To explore the influence of structural and subjective variables—such as household composition, functional dependency, access to municipal services, and satisfaction with social relationships—on the perception of loneliness among older adults in rural areas.

### 2.1 Research hypotheses

Based on the proposed objectives and the Cognitive Discrepancy Model, the following hypotheses are formulated:

- H1: Older adults who live alone exhibit significantly higher levels of subjective loneliness compared to those who live with others.
- H2: Frequent participation in personal activities (such as reading, sewing, talking on the phone, or doing household tasks) and community activities (such as going out, exercising, participating in social events, or maintaining in-person contact) is associated with lower levels of perceived loneliness.
- H3: The perception of limited access to municipal services and the presence of functional dependency are associated with a higher prevalence of subjective loneliness.

## 3 Methodology

### 3.1 Participants

The study employed a non-probabilistic cluster sampling design, focusing on five strategically selected Social Action Zones (ZAS) in the province of Salamanca. The selection of these zones was based on technical and empirical criteria: high depopulation (<10 inhabitants/km^2^), an ageing rate equal to or greater than 50%, and low physical, digital, and social accessibility. Participant selection followed structural and functional heterogeneity criteria (age, sex, living arrangements, dependency, and use of social services), with the aim of capturing a diverse range of profiles within the exploratory framework of the study.

The study population lives in rural municipalities with 500 or fewer inhabitants, located in areas with known risk factors that increase the likelihood of experiencing feelings of loneliness. These areas are characterized by severe depopulation, poor accessibility, a high aging rate, and predominantly small household units composed of one or two members.

The sample selection followed several stages. First, the Social Action Zones (Zonas de Acción Social) where data collection would take place were chosen based on three criteria: (1) very low population density (fewer than 10 inhabitants per km^2^); (2) poor accessibility, physical (transport and communication), digital (internet), and social (services); and (3) a high aging index, equal to or greater than 50%. Based on these criteria, five zones in the western province of Salamanca were selected: Ciudad Rodrigo II, Linares, Lumbrales, Tamames, and Vitigudino. These zones include second-tier regional centers (Vitigudino and Lumbrales) and key territorial nuclei (Tamames, Linares, Fuenteguinaldo, and Fuentes de Oñoro).

Second, municipalities with populations of 0–250 and 251–500 were selected, maintaining the existing ratio of 70/30 found in these rural areas, as aging is more pronounced in the smallest municipalities. Accordingly, 70% of the sample came from municipalities with 0–250 inhabitants, and 30% from those with 251–500.

Third, to ensure sample representativeness, five criteria were applied in participant selection:

1. Age range; the three age brackets (60–69, 70–79, and 80+) were represented proportionally to the actual rural population.
2. Sex, accounting for the masculinization of the adult rural population and the feminization of old age, a higher proportion of men was included in the 60–69 and 70–79 age groups, and more women in the 80+ group.
3. Household composition, participants from households of one or two members were included;
4. Dependency, households with at least one member officially recognized as dependent were included;
5. Use of social services provided by the Salamanca Provincial Council; participants who used and did not use the available services were included.”

The final sample comprised 153 participants, residing in municipalities of 0–250 inhabitants (56.8%) and 251–500 (43.2%). Women made up 62.9% of the sample, with an average age of 83.07 years (SD = 8.12), ranging from 60 to 98 years. A total of 75.7% were functionally literate (able to read and write), 81.6% were pensioners, and 87.8% had monthly incomes below 1,000 euros. Nearly half (49%) were married or partnered and had an average of 2 children (range: 0–8). About 32.7% lived alone, most of them (55.2%) for more than 5 years, and primarily due to the loss of a spouse or partner (73.5%). Of those living with someone (67.3%), 75.2% cohabited with a spouse or partner. A total of 47.7% had an officially recognized degree of dependency; 38.6% used home help services, and 21.4% used tele-assistance. See results in Table 1.

**TABLE 1:** Descriptive statistics of the sociodemographic characteristics of the sample (N = 153).

Participants’ characteristics	n	% Valid
Population of residence	125	​
0–250 inhabitants	71	56.8
251–500 inhabitants	54	43.2
Age	M = 83.07; DT = 8.12; Min = 60; Max = 98	
Gender	151	151
Male	56	56
Female	95	95
Level of education	152	152
Cannot read and write	6	6
Can read and write	115	115
Primary and secondary	30	30
University	1	1
Employment status	152	​
Employed	1	0.7
Pensioner	124	81.6
Retired without pension	25	16.4
Others	2	1.3
Monthly income level (euros)	139	​
Less than 1000	122	87.8
Between 1000–2000	17	12.2
More than 2000	0	0.0
Marital status	153	​
Married or cohabiting	75	49
Others (divorced or separated, widowed, single)	78	51
N. Of children	M = 2.44; DT = 1.99; Min = 0; Max = 8	
Lives alone	49	32.7
Time	​	​
From 1 year ago	3	10.3
2–5 years	10	34.5
6–10 years	4	13.8
More than 10 years	12	41.4
Reason	​	​
By choice	9	18.4
By loss of spouse/Partner	36	73.5
By loss of parent	3	6.1
By loss of descendants	1	2
Live in company	101	67.3
Spouse/Partner	76	75.2
Father/Mother	4	4.0
Children	13	12.9
Others	8	7.9
Recognized dependency	76	75.2
Use of social services	​	​
Home care	54	38.6
Telecare	31	21,4

### 3.2 Variables and instruments

The instrument used was a structured questionnaire administered in interview format, composed of five distinct sections that include both objective and subjective variables relevant to the study of loneliness among older adults in rural settings.

- Sociodemographic Questionnaire. This section includes items aimed at collecting basic information on participants’ personal characteristics, such as age, gender, marital status, number of children, educational level, employment status, place of residence (municipality), type of dwelling, income level, household composition (living alone: reason, duration, intention to continue living alone).

It also included the official assessment of dependency status, specifying Degree I, II, or III, according to the current scale established under the Spanish Law for the Promotion of Personal Autonomy and Care for Dependent Persons. Additionally, the Barthel Index was applied, an extensively used tool for assessing functional capacity in basic daily living activities such as hygiene, mobility, eating, or continence. This index provides a quantitative score to determine the level of autonomy or need for support and was used as a structural variable in the analysis of loneliness.

- Use of Social Services and Support. This section explores access to and use of community resources available in the municipality, such as home help services, tele-assistance, day centers, nursing homes, pharmacies, shops, public transportation, religious services, internet access, local associations, and financial services. It also records whether the person provides or receives care, allowing for analysis of the balance between support given and received, and its potential relationship with loneliness.
- Leisure Activities at Home. Participants were asked about the regular performance of activities such as watching television, listening to the radio, reading, cooking, doing crosswords, using a computer, sewing, gardening, DIY, caring for pets, or playing board games. These activities were recorded in a dichotomous format (yes/no), allowing for an assessment of personal engagement, domestic autonomy, and the symbolic or emotional connectedness provided by pastimes.
- Participation in Community Activities. This section includes a scale measuring frequency and satisfaction with participation in out-of-home activities, such as going for walks, visiting family and friends, attending social or religious events, volunteering, support groups, workshops, or civic activities. Each activity was assessed in terms of weekly frequency (none, 1–2 days, 3–4 days, 5–6 days, 7 days) and whether the level of participation was perceived as sufficient, insufficient, or excessive. This section evaluates the relational dimension of the social environment and its role in mitigating feelings of loneliness.
- UCLA Loneliness Scale Version 3. Perceived loneliness was assessed using the Spanish-adapted version of the UCLA Loneliness Scale (Version 3), validated for older adults in Spain (Velarde-Mayol et al., 2015). It consists of 10 items rated on a 4-point Likert scale (1 = always, 2 = often, 3 = rarely, 4 = never), yielding a total score between 10 and 40, where lower scores indicate higher levels of loneliness. The scale includes three dimensions: social isolation, emotional loneliness, and social connectedness. It shows excellent psychometric properties, with high internal consistency (α = 0.95), making it a suitable tool for evaluating subjective loneliness in older adults.

### 3.3 Procedure

The research team contacted the Provincial Council of Salamanca to gather information on the province’s Social Action Zones, with the aim of selecting those most distant from the capital, with higher rates of depopulation and aging, and fewer services (communication, health, financial, commercial, etc.).

Once the Social Action Zones and municipalities were selected based on the defined criteria, data collection was carried out by the social workers assigned to these areas, who had previously received training from the research team. The fieldwork was conducted between January and May 2024.

The questionnaire and the loneliness scale were administered in individual face-to-face interviews, following the participants’ informed consent, after they had been informed about the study’s objectives and assured of the anonymity of their responses.

The study received approval from the Bioethics Committee of the University of Salamanca, under registration number 405.

### 3.4 Statistical analysis

First, descriptive statistics were calculated for the sociodemographic variables. Frequencies and percentages were obtained for categorical variables and means and standard deviations for quantitative variables.

Second, to examine the relationship between levels of loneliness and study variables, chi-square tests were conducted using contingency tables. Participants’ scores on the UCLA Loneliness Scale were categorized into three levels: normal, moderate, and severe, following the cutoff points established in the manual (Velarde-Mayol et al., 2015). Subsequently, due to the low number of cases in the severe category, the moderate and severe levels were grouped together for analysis.

For the same reason, some categories of sociodemographic variables (educational level, marital status, reason for living alone) were grouped, while others were excluded due to lack of representation—for example, income categories and some community activities under the frequency dimension (caring for children or loved ones, learning activities, volunteering, religious activities, support groups, participation in associations, civic or political activities). Similarly, some services (internet access, nursing home, and day center) and at-home leisure activities (computer use) were not analyzed due to a low number of cases.

Statistical analyses were performed using SPSS v.26, with a significance level set at α = 0.05.

### 3.5 Results

The sample, selected from municipalities with fewer than 500 inhabitants had an average age of 88 years, consisted of retired individuals with monthly incomes under €2000, and lived in households composed of one or two people. Among them, 9.8% showed severe levels of loneliness, 31.4% moderate, and 58.8% normal levels.

#### 3.5.1 Relationship between levels of loneliness and sociodemographic characteristics and municipal services

A higher percentage of participants reporting moderate or severe levels of loneliness were those living in municipalities with 251–500 inhabitants, women, individuals who could not read or had basic literacy, those with monthly incomes between €1000 and €2000, individuals who were not married or in a partnership, those who lived alone due to the loss of a spouse, partner, or family member, those who were functionally dependent, and those who did not use home help or tele-assistance services.

However, the only statistically significant relationship was found between household size and loneliness levels: the percentage of individuals with moderate or severe loneliness was significantly higher among those living alone compared to those living with others (see Table 2).

**TABLE 2:** Frequencies and percentages of participants with moderate or severe levels of loneliness across different categories of sociodemographic variables, and significance test results.

Sociodemographic variables	Level of loneliness moderate-severe n (% valid)	χ2, p
Population of residence		
0–250 inhabitants	21 (29.6)	χ2 (1, N = 125) = 3.69, p = 0.055
251–500 inhabitants	25 (46.3)	
Gender		
Male	22 (39.3)	χ2 (1, N = 151) = 0.22, p = 0.641
Female	41 (43.2)	
Level of education		
Can’t read and write/Can read and write	54 (44.6)	χ2 (1, N = 152) = 2.47, p = 0.116
Primary, secondary or university	9 (29.0)	
Level of monthly income (euros)		
Less than 1000	50 (41.0)	χ2 (1, N = 139) = 1.94, p = 0.164
Between 1000–2000	10 (58.8)	
Marital status		
Married or in couple	29 (38.7)	χ2 (1, N = 153) = 0.38, p = 0.536
Others (divorced or separated, widowed, single)	34 (43.6)	
Family unit		
2 members	34 (33.7)	χ2 (1, N = 150) = 6,29, p = 0.012
1 member	27 (55.1)	
Reason for living alone		
Choice	4 (44.4)	χ2 (1, N = 49) = 0.51, p = 0.477
Loss (spouse/partner, parents, descendants)	23 (57.5)	
Dependency		
No	28 (36.8)	χ2 (1, N = 149) = 1.08, p = 0.292
Yes	33 (45.2)	
Use of social services		
Home help		
No	38 (44.2)	χ2 (1, N = 140) = 1.11, p = 0.291
Yes	19 (35.2)	
Telecare		
No	50 (43.9)	χ2 (1, N = 145) = 0.26, p = 0.607
Yes	12 (38.7)	

Source: Own elaboration.

Regarding the services available to participants in their municipalities, the most common were mobile vendors, pharmacies or first-aid dispensaries, religious services, and local shops. In contrast, day centers, residential facilities, and internet access were virtually nonexistent. Notably, there was also a surprisingly low percentage of availability of bars and transportation services.

In general, a higher percentage of participants with severe or moderate levels of loneliness was observed among those living in municipalities without these services. However, no statistically significant associations were found between loneliness levels and the availability of these services in the municipality (see Table 3).

**TABLE 3:** Frequencies and percentages of participants who had access to each service, and frequencies and percentages of those with moderate or severe loneliness depending on whether or not they had access, with significance test results.

Services	Available	Level of loneliness moderate-severe n (% valid)		χ2, p
		They have the service		
	n (% valid)	Yes	No	
Peddling	89 (65,9)	35 (39.3)	20 (43.5)	χ2 (1, N = 135) = 0.22, p = 0.642
Pharmacy/Pharmacy	85 (65,9)	39 (45.9)	13 (29.5)	χ2 (1, N = 129) = 3.22, p = 0.073
Religious services	87 (60,0)	36 (41.4)	25 (43.1)	χ2 (1, N = 145) = 0.04, p = 0.837
Shops	61 (51,3)	28 (45.9)	18 (31.0)	χ2 (1, N = 119) = 2.77, p = 0.140
Associations	39 (30,5)	13 (33.3)	37 (41.6)	χ2 (1, N = 128) = 0.77, p = 0.379
Bar	34 (28,1)	12 (35.3)	37 (42.5)	χ2 (1, N = 121) = 0.53, p = 0.466
Transportation	33 (27,5)	10 (30.3)	33 (37.9)	χ2 (1, N = 120) = 0.61, p = 0.437
Bank/Financial agent	23 (22,8)	10 (43.5)	27 (34.6)	χ2 (1, N = 101) = 0.60, p = 0.438
Workshops	19 (20,4)	5 (26.3)	26 (35.1)	χ2 (1, N = 93) = 0.53, p = 0.467
Internet access	7 (6,9)	​	​	​
Residence	3 (3,2)	​	​	​
Day care center	2 (2,5)	​	​	​

Source: Own elaboration.

#### 3.5.2 Relationship between loneliness levels and in-home and community-based leisure activities

Watching television is the most common leisure activity among participants (85.6%), while almost no one uses a computer for entertainment (0.7%). In nearly all cases, except for pet care, the percentage of participants with severe or moderate loneliness is higher among those who do not engage in these at-home activities. However, loneliness levels are only significantly associated with the activities of talking on the phone, reading, and sewing. The percentage of participants who engage in these activities and report moderate or severe loneliness is significantly lower than among those who do not engage in them (see Table 4).

**TABLE 4:** Frequencies and percentages of participants who engage in these activities, those with moderate or severe loneliness among them, and significance test results.

Home entertainment	Perform	Level of loneliness moderate-severe n (% valid)		χ2,p
		Perform		
		Yes	Yes	
Watching TV	131 (85,6)	53 (40.5)	10 (45.5)	χ2 (1, N = 153) = 0.19, p = 0.659
Talking on the phone	60 (39,2)	18 (30.0)	45 (48.4)	χ2 (1, N = 153) = 5.09, p = 0.024
Reading	52 (34,0)	14 (26.9)	49 (48.5)	χ2 (1, N = 153) = 6.61, p = 0.010
Cooking	47 (30,7)	17 (36.2)	46 (43.4)	χ2 (1, N = 153) = 0.70, p = 0.402
Crossword puzzles	35 (22,9)	11 (31.4)	52 (44.1)	χ2 (1, N = 153) = 1.78, p = 0.182
Caring for pets	35 (22,9)	15 (42.9)	48 (40.7)	χ2 (1, N = 153) = 0.05, p = 0.818
Gardening	34 (22,2)	10 (29.4)	53 (44.5)	χ2 (1, N = 153) = 2.50, p = 0.114
Listening to the radio	32 (20,9)	10 (31.3)	53 (43.8)	χ2 (1, N = 153) = 1.65, p = 0.199
Sewing	26 (17,0)	6 (23.1)	57 (44.9)	χ2 (1, N = 153) = 4.24, p = 0.040
Playing cards/Board games	21 (13,7)	7 (33.3)	56 (42.4)	χ2 (1, N = 151) = 0.62, p = 0.432
Do-it-yourself/Handicrafts	17 (11,1)	4 (23.5)	59 (43.4)	χ2 (1, N = 153) = 2.46, p = 0.117
Computer	1 (0,7)	​	​	​

Source: Own elaboration.

It is important to note that, while more than 30% of participants engage in talking on the phone and reading, only 17% report engaging in sewing.

#### 3.5.3 Relationship between loneliness levels and community activities

In relation to community activities, in most cases, except for volunteering and support groups, the percentage of participants with moderate or severe levels of loneliness is higher among those who never engage in these activities and among those who are dissatisfied with the frequency at which they do engage.

Significant relationships were found between levels of loneliness and both the frequency and satisfaction of participation in the following activities:

On the one hand, for activities such as going out and doing exercise or sports, the percentage of participants with moderate or severe levels of loneliness is significantly higher among those who do not perform these activities on any day of the week and among those who consider the frequency of participation insufficient. It is noteworthy that, in the case of going out, the percentage of participants with moderate or severe levels of loneliness is significantly lower only when the activity is performed daily.

On the other hand, regarding activities that involve being with family, friends, or a significant other, a significantly higher percentage of participants with moderate or severe levels of loneliness was found among those who do not engage in these activities at all during the week. Furthermore, satisfaction with the frequency of engagement also showed a significant relationship: the percentage of participants with moderate or severe levels of loneliness is significantly lower among those who consider the frequency sufficient (see Table 5).

**TABLE 5:** Frequencies and percentages of participants with moderate and severe levels of loneliness according to the frequency of community activities and their satisfaction with them, and the corresponding statistical significance tests.

Community activities	Level of loneliness moderate-severe			χ2, p	Level of loneliness moderate-severe		χ2, p
	n (% valid)				n (% valid)		
	None	Some days	Every day		Not enough	Sufficient	
Hanging out	12 (66.7) Res. Tip. Corr = 2.3	17 (60.7) Res. Tip. Corr = 2.3	34 (32.1) Res. Tip. Corr = −3.6	χ2 (2, N = 152) = 12.84, p = 0.002	24 (63.2)	36 (32.7)	χ2 (1, N = 148) = 10.85, p = 0.001
Being with family	12 (63.2%) Res. Tip. Corr = 2.1	28 (41.8) Res. Tip. Corr = 0.2	22 (33.8) Res. Tip. Corr = −1.6	χ2 (2, N = 151) = 5.25, p = 0.073	37 (54.4)	24 (30.4)	χ2 (1, N = 147) = 9.42, p = 0.009
Contact with family	4 (26.7) Res. Tip. Corr = −1.2	30 (45.5) Res. Tip. Corr = 1.0	28 (39.4) Res. Tip. Corr = −0.3	χ2 (2, N = 152) = 1.89, p = 0.389	14 (58.3)	48 (38.1)	χ2 (1, N = 150) = 3.41, p = 0.065
Being with friends	22 (53.7) Res. Tip. Corr = 2.0	28 (38.9) Res. Tip. Corr = −0.4	11 (28.9) Res. Tip. Corr = −1.7	χ2 (2, N = 151) = 5.13, p = 0.077	33 (55.9)	28 (31.8)	χ2 (1, N = 147) = 8.46, p = 0.004
Contact by phone or internet	47 (43.9) Res. Tip. Corr = 1.4	11 (32.4) Res. Tip. Corr = −1.1	3 (30.0) Res. Tip. Corr = −0.7	χ2 (2, N = 151) = 1.92, p = 0.384	24 (51.1)	34 (35.1)	χ2 (1, N = 144) = 3.38, p = 0.066
Going to the bar/Eating out	45 (46.9) Res. Tip. Corr = 2.0	13 (31.0) Res. Tip. Corr = −1.5	4 (28.6) Res. Tip. Corr = −1.0	χ2 (2, N = 152) = 4.02, p = 0.134	26 (49.1)	30 (33.0)	χ2 (1, N = 144) = 3.65, p = 0.056
Being with someone special	33 (51.6) Res. Tip. Corr = 2.2	12 (36.4) Res. Tip. Corr = −0.7	12 (30.0) Res. Tip. Corr = −1.8	χ2 (2, N = 137) = 5.20, p = 0.074	25 (67.6)	30 (31.9.9)	2 (1, N = 131) = 13.86 p <0 .001
Caring for children or loved one	54 (46.2) Res. Tip. Corr = 1.8	​	8 (27.6) Res. Tip. Corr = −1.8	χ2 (1, N = 146) = 3.28, p = 0.070	13 (59.1)	41 (36.9)	χ2 (1, N = 133) = 3.74 p = 0.053
Learning activities	57 (44.5) Res. Tip. Corr = 1.7	6 (26.1) Res. Tip. Corr = −1.7	​	χ2 (1, N = 151) = 2.73, p = 0.099	23 (44.2)	35 (39.8)	χ2 (1, N = 140) = 0.27, p = 0.605
Volunteering	58 (41.1) Res. Tip. Corr = −0.4	2 (50.0) Res. Tip. Corr = 0.4	​	χ2 (1, N = 145) = 0.13, p = 0.723	13 (48.1)	37 (37.4)	2 (1, N = 151) = 1.03, p = 0.310
Religious activities	30 (44.1) Res. Tip. Corr = 0.6	33 (39.7) Res. Tip. Corr = −0.6	​	χ2 (1, N = 152) = 0.36, p = 0.548	27 (45.8)	33 (37.1)	χ2 (1, N = 148) = 1.11 p = 0.292
Support groups	58 (42.3) Res. Tip. Corr = −0.8	4 (57.1) Res. Tip. Corr = 0.8	​	χ2 (1, N = 144) = 0.60, p = 0.440	20 (52.6)	34 (37.4)	χ2 (1, N = 129) = 2.57, p = 0.109
Hobbies/Leisure	49 (45.4) Res. Tip. Corr = 1.5	11 (34.4) Res. Tip. Corr = −0.9	2 (22.2) Res. Tip. Corr = −1.2	χ2 (2, N = 149) = 2.71, p = 0.258	31 (49.2)	27 (33.8)	χ2 (1, N = 143) = 3.49, p = 0.062
Going to sporting events/	60 (43.2) Res. Tip. Corr = 1.7	0 (0.0) Res. Tip. Corr = −1.7	​	χ2 (1, N = 143) = 2.98, p = 0.085	25 (53.2)	31 (34.1)	χ2 (1, N = 138) = 4.70, p = 0.030
​	41 (53.2) Res. Tip. Corr = 3.0	6 (25.0) Res. Tip. Corr = −1.8	16 (31.4) Res. Tip. Corr = −1.8	χ2 (2, N = 152) = 9.23, p = 0.010	23 (60.5)	37 (33.9)	χ2 (1, N = 147) = 8.24, p = 0.004
Exercise/	51 (43.6) Res. Tip. Corr = 0.6	12 (37.5) Res. Tip. Corr = −0.6	​	χ2 (1, N = 149) = 0.38, p = 0.537	22 (41.5)	36 (39.6)	χ2 (1, N = 144) = 0.05, p = 0.818
Sport	61 (41.5) Res. Tip. Corr = 0.3	1 (33.3) Res. Tip. Corr = −0.3	​	χ2 (1, N = 150) = 0.08, p = 0.776	14 (45.2)	41 (38.3)	χ2 (1, N = 138) = 0.47, p = 0.493

## 4 Discussion

The findings of this study reinforce the understanding of loneliness as a multidimensional phenomenon, profoundly shaped by the interaction of personal, contextual, and social variables. In this regard, the Cognitive Discrepancy Model (Peplau and Perlman, 1979) provides a particularly useful theoretical framework for interpreting the results, as it conceptualizes loneliness as the result of a gap between desired and perceived social relationships.

In interpreting the results, the Cognitive Discrepancy Model not only serves as a general conceptual framework, but also substantively structures the analysis of factors associated with loneliness. For instance, variables such as widowhood, lack of meaningful interaction, or limited community participation directly impact the gap between desired and actual social relationships—the central axis of the model. Moreover, the fact that the availability of services is not associated with lower levels of loneliness suggests that structural resources alone do not foster wellbeing unless they translate into experiences of emotional connection and belonging. In this regard, the empirical findings reinforce the validity of the cognitive-subjective approach proposed by Peplau and Perlman, and its relevance in rural contexts marked by multiple vulnerabilities.

Although this study employed the UCLA Loneliness Scale v3 due to its strong psychometric properties and alignment with the theoretical framework of cognitive discrepancy, we acknowledge the value of other tools such as the ESTE II Social Loneliness Scale (Pinel Zafra et al., 2009), developed within the Spanish context. This scale includes dimensions related to perceived social support, community participation, and the use of technology, especially relevant in community-based interventions. While it was not directly applied in this study, our methodological design did capture these dimensions through various complementary instruments and structured interviews. We consider its inclusion appropriate for future studies aiming for a more comprehensive assessment of loneliness, incorporating both its subjective and social dimensions.

Hypothesis H1 is partially confirmed, as a significant association was observed between living alone and higher levels of loneliness, particularly in cases of widowhood. This finding is consistent with previous research that highlights the loss of emotional bonds, such as the death of a partner or estrangement from children, as one of the key triggers of loneliness within the Cognitive Discrepancy Model (Peplau and Perlman, 1979; Dahlberg et al., 2018). Although no statistically significant associations were found with other sociodemographic variables in this sample, their theoretical and empirical relevance warrants attention.

Firstly, gender remains a critical structural determinant. Women make up the majority in older age groups due to their longer life expectancy, but they also face a higher likelihood of widowhood, lower contributory pensions, and life trajectories marked by informal labor and caregiving roles. All these factors contribute to what is known as the feminization of poverty (Béland and Irwin, 2021), a condition that increases emotional, social, and economic vulnerability in old age. Additionally, women have been found to be more likely to express emotions related to distress (Borys and Perlman, 1985), which could influence both the experience and detection of loneliness.

Secondly, advanced age is widely recognized in the literature as a predictor of loneliness due to multiple cumulative causes: physical decline, bereavement, reduced social networks, and withdrawal from public life (Wagner et al., 1996; Lang et al., 1998). Although this study did not find significant differences between age groups, the sample had a very high average age, suggesting potential homogeneity that may have masked internal differences.

Educational level and income have also been consistently linked to greater or lesser access to protective networks and resources. Individuals with lower levels of education tend to face more difficulties integrating into community dynamics that require cognitive, digital, or communication skills, which can reinforce feelings of exclusion (Cardona et al., 2013). In rural settings, this educational gap is further exacerbated by the scarcity of lifelong learning opportunities, particularly for older women.

Marital status also influences the experience of loneliness. Various studies have shown that living with a partner acts as a buffer against isolation (Aartsen and Jylhä, 2011; Dahlberg et al., 2016). In this study’s sample, widowed individuals or those living alone due to loss report higher levels of loneliness compared to those who cohabit with someone. However, the quality of cohabitation was not assessed in this study, which may be an important factor for future research.

In sum, sociodemographic factors should not be analyzed in isolation, but rather as interactive variables that shape differentiated life trajectories. An intersectional perspective helps to understand how the combination of age, gender, social class, and rurality can lead to accumulated inequalities that intensify the experience of loneliness. This approach is essential for designing interventions that are sensitive to the biographical and contextual specificities of older adults in rural areas.

Regarding Hypothesis H2, the data indicate that certain personal activities, such as reading, talking on the phone, or sewing, act as protective factors. The finding that even symbolic or mediated interactions can alleviate perceptions of loneliness offers an important nuance to current discussions on active ageing (Cacioppo and Patrick, 2008; Niu et al., 2017). In rural environments, where face-to-face contact may be hindered by mobility or distance barriers, such activities become particularly valuable by fostering emotional connection to the social environment, even indirectly.

With respect to community activities, the results show a clear relationship between the frequency/satisfaction with activities like going out, exercising, or spending time with loved ones, and lower levels of loneliness. Notably, in the case of “going out,” only daily engagement is associated with a significant reduction in loneliness. This finding underscores the importance of both the regularity and perceived quality of social interactions, core aspects of the Cognitive Discrepancy Model and recent literature on subjective wellbeing in later life (Schwanen and Wang, 2014).

However, Hypothesis H3 was not confirmed. Although participants lacking access to basic services tended to report higher levels of loneliness, no statistically significant associations were found between the availability of these services in the municipality and perceived loneliness. This result may be explained by non-structural barriers such as lack of transportation, perceived insecurity, or emotional disconnection from available services (Rantakokko et al., 2013; Organización Mundial de la Salud, 2015). Literature suggests that the mere existence of resources does not ensure effective use if factors such as symbolic accessibility, institutional trust, or cultural relevance are not addressed (Golant, 2011).

Taken together, the findings encourage a move beyond a purely structural approach to addressing loneliness. It is not enough to offer community activities or services; these initiatives must foster meaningful social bonds. The most effective interventions will be those that connect older adults with other people, not merely with activities. This aligns with the findings of De Jong Gierveld and Tesch-Römer (2012), who emphasize the primacy of relational dimensions over mere time use.

Finally, these results underscore the need to adopt an ecological and intersectional approach in the design of public policy, as suggested by the ICF-WHO framework (2002). The experience of loneliness is shaped by personal factors (gender, functionality, life course), environmental factors (accessibility, transportation, safety), and social factors (type and quality of relationships). Effective interventions must account for this complexity and foster environments that facilitate human connection beyond the mere provision of formal services. The evidence gathered in this study indicates that the key to reducing loneliness lies not so much in participation *per se*, but in the ability to establish meaningful contact with others. In this regard, any programmatic design, whether from public administration, community organizations, or the health sector, must recognize that social interaction is not ancillary, but a central axis of well-being in later life. Loneliness is not fought with occupation, but with connection.

## 5 Study limitations

Despite the methodological rigor and contextual relevance of the findings, this study presents several limitations that should be considered when interpreting the results.

First, the sample size (N = 153) and its regional focus limit the generalizability of the findings to other rural populations with different demographic, geographic, or cultural characteristics. The territorial concentration in five areas of western Salamanca provides local depth but reduces comparative breadth.

Second, the need to group categories in certain variables, such as educational level, marital status, or community activities, to allow for statistical analysis may have limited the ability to detect more nuanced differences. Likewise, the exclusion of variables with low representation affected the interpretive richness in certain sections, such as internet access or use of less common services.

Third, the cross-sectional nature of the design prevents causal relationships from being established between the variables analyzed. Although significant associations between certain factors and levels of loneliness were identified, these relationships cannot be assumed to be unidirectional or stable over time.

It is also important to highlight the possible influence of social desirability. Since the data were collected through face-to-face interviews conducted by social workers in an institutional setting, it is likely that some participants offered more socially acceptable responses or minimized their feelings of loneliness out of modesty, trust, or fear of stigmatization. This may have led to an underestimation bias in the main variable.

Lastly, although the instrument used to measure loneliness (UCLA-3) has excellent psychometric properties and is widely recognized internationally, the study did not include specific tools developed within the Spanish context that allow for a more community-oriented assessment of loneliness, such as the ESTE II Social Loneliness Scale (Pinel et al., 2009). However, the study’s methodological design did include variables aligned with that scale, such as perceived social support, community participation, and the use of technology, through other complementary questionnaires. We believe that future research could benefit from the combined use of both instruments to achieve a more comprehensive evaluation of loneliness, integrating both its subjective and social dimensions.

Despite these limitations, this study provides a rigorous and context-sensitive empirical approach to understanding loneliness among older adults living in highly depopulated rural areas—an issue that remains underexplored in the scientific literature. The combination of a validated scale, field-based data collection, and the inclusion of sociodemographic, personal, and community variables gives the study a solid foundation for guiding future interventions that are sensitive to local contexts and the life trajectories of older adults.

Future studies could address these limitations through longitudinal designs, mixed methods (quantitative and qualitative), and larger samples that allow for comparisons across diverse rural settings and refinement in identifying protective and risk factors.

## 6 Conclusion

This study confirms that loneliness among older adults in rural areas is a complex phenomenon, influenced by the interaction between personal, social, and contextual variables. The findings support the relevance of the Cognitive Discrepancy Model (Peplau and Perlman, 1979) as an explanatory framework, highlighting the importance of subjective expectations and evaluations in shaping the experience of loneliness.

Among the main findings, living alone emerged as a significant risk factor, especially when associated with emotional loss such as widowhood. It was also confirmed that certain personal and community activities, such as reading, talking on the phone, exercising, or maintaining regular contact with family, have a protective effect, emphasizing the central role of emotional, symbolic, and social connection in the subjective wellbeing of older adults.

However, no statistically significant associations were found between the availability of municipal services and levels of loneliness, suggesting that formal access to resources alone is not sufficient to mitigate isolation. This finding calls for a rethinking of social policies that combine structural and subjective approaches, and functional and emotional dimensions.

The study also highlights that sociodemographic factors should not be interpreted in isolation. Variables such as gender, age, educational level, or income interact in differentiated life trajectories that generate unequal conditions of vulnerability. The feminization of poverty in old age, the digital divide, functional dependency, and territorial disconnection are elements that form a map of accumulated risks requiring intersectional responses.

Overall, the results presented here invite a revision of traditional approaches to aging from a more holistic perspective, one focused on human connection, a sense of belonging, and the construction of emotionally meaningful environments.

Future policies and research should move toward intervention models that simultaneously address both the subjective and social dimensions of loneliness, promoting not only access to resources, but above all, the creation of meaningful connections that provide a sense of purpose, security, and companionship.

## 7 Implications for intervention and public policy design

The findings of this study suggest the need to rethink current strategies for preventing and addressing loneliness among older adults in rural areas. First and foremost, interventions must acknowledge that loneliness is not merely a structural phenomenon associated with a lack of services or geographic isolation, but a deeply subjective experience shaped by relational, emotional, and symbolic factors. In this regard, public policies should prioritize the strengthening of meaningful social bonds, going beyond mere access to resources or activities.

Given the key role of living alone, especially following widowhood, it is urgent to develop emotional support systems and relational accompaniment strategies that go beyond assistance-based approaches. Initiatives such as informal in-home companionship, intergenerational mentoring programs, or neighborhood care networks could serve as effective tools to reduce emotional isolation. These measures should take into account the reality of the feminization of rural aging, adapting their design to the biographical trajectories, gender roles, and material conditions of older women.

Moreover, the protective value of certain personal activities such as reading, talking on the phone, or sewing highlights the importance of promoting non-face-to-face yet emotionally meaningful forms of engagement. Active aging programs should integrate these elements, providing accessible resources for their practice, especially in households without access to digital technologies. On the other hand, the low correlation between available municipal services and levels of loneliness indicates that offering resources is not enough: it is essential to promote symbolic and emotional appropriation of these services through proximity strategies, community mediation, and co-designed participation.

From an ecological perspective, loneliness-reduction policies must be sensitive to the interaction between personal (age, gender, dependency), social (quality of relationships), and territorial (accessibility, transportation, safety) dimensions. This implies acting not only on the physical environment but also on the perceptions, emotions, and expectations that older adults hold regarding their social ties. The most effective solutions will be those that foster a sense of belonging, dignity, and social usefulness.

Ultimately, a paradigm shift is necessary, from an approach focused on time occupation or service provision to one rooted in community-building and the strengthening of relational networks. Loneliness cannot be resolved through programs or activities alone, but through close, sustainable, and meaningful bonds. Therefore, it is recommended that rural public policies incorporate qualitative assessment tools, participatory methodologies, and an intersectional perspective to ensure that institutional responses align with the real, felt, and expressed needs of older adults themselves.

## Data Availability

The original contributions presented in the study are included in the article/supplementary material, further inquiries can be directed to the corresponding author.
